# Effects of high frequency rTMS of contralesional dorsal premotor cortex in severe subcortical chronic stroke: protocol of a randomized controlled trial with multimodal neuroimaging assessments

**DOI:** 10.1186/s12883-022-02629-x

**Published:** 2022-04-01

**Authors:** Jiali Li, Hewei Wang, Yujian Yuan, Yunhui Fan, Fan Liu, Jingjing Zhu, Qing Xu, Lan Chen, Miao Guo, Zhaoying Ji, Yun Chen, Qiurong Yu, Tianhao Gao, Yan Hua, Mingxia Fan, Limin Sun

**Affiliations:** 1 Huashan Hospital, Fudan University, Shanghai, China; 2 East China Normal University, Shanghai, China; 3 The Third Rehabilitation Hospital, Shanghai, China

**Keywords:** Stroke, Hemiplegia, Transcranial magnetic stimulation, Dorsal premotor cortex, Functional MRI

## Abstract

**Background:**

Previous studies have revealed that low frequency repeated transcranial magnetic stimulation (rTMS) on the contralesional primary motor cortex (cM1) is less effective in severe stroke patients with poor neural structural reserve than in patients with highly reserved descending motor pathway. This may be attributed to the fact that secondary motor cortex, especially contralesional dorsal premotor cortex (cPMd), might play an important compensatory role in the motor function recovery of severely affected upper extremity. The main purpose of this study is to compare the effectiveness of low frequency rTMS on cM1 and high frequency rTMS on cPMd in subcortical chronic stroke patients with severe hemiplegia. By longitudinal analysis of multimodal neuroimaging data, we hope to elucidate the possible mechanism of brain reorganization following different treatment regimens of rTMS therapy, and to determine the cut-off of stimulation strategy selection based on the degree of neural structural reserve.

**Methods/design:**

The study will be a single-blinded randomized controlled trial involving a total of 60 subcortical chronic stroke patients with severe upper limb motor impairments. All patients will receive 3 weeks of conventional rehabilitation treatment, while they will be divided into three groups and receive different rTMS treatments: cM1 low frequency rTMS (*n* = 20), cPMd high frequency rTMS (*n* = 20), and sham stimulation group (*n* = 20). Clinical functional assessment, multimodal functional MRI (fMRI) scanning, and electrophysiological measurement will be performed before intervention, 3 weeks after intervention, and 4 weeks after the treatment, respectively.

**Discussion:**

This will be the first study to compare the effects of low-frequency rTMS of cM1 and high-frequency rTMS of cPMd. The outcome of this study will provide a theoretical basis for clarifying the bimodal balance-recovery model of stroke, and provide a strategy for individualized rTMS treatment for stroke in future studies and clinical practice.

**Trial registration:**

Chinese Clinical Trial Registry, ChiCTR1900027399. Registered on 12 Nov 2019, http://www.chictr.org.cn/showproj.aspx?proj=43686.

## Background

Hemiplegia is one of the most common functional defects after stroke. Although stroke patients can recover spontaneously, most patients in the chronic phase will still suffer from upper limb dysfunction, especially hand dysfunction. It seriously affects the patients’ abilities of daily living, and imposes a heavy burden on their families and the society [1].

Transcranial magnetic stimulation (TMS), one of the non-invasive brain stimulation (NIBS) techniques, have been widely used in research and clinical treatment to promote the recovery of motor function in stroke patients due to its safety, noninvasiveness, and painlessness [2, 3]. Repeated TMS (rTMS) is a commonly used stimulation mode developed on the basis of TMS. High frequency rTMS (≥1 Hz) has an excitatory effect on cortex neurons, whereas low frequency rTMS (< 1 Hz) inhibits the activity of cortex neurons [4–6]. In recent years, rTMS has shown a great potential in stroke rehabilitation, with many studies reporting that it can improve the motor function of stroke patients with hemiplegia [7–10]. However, there are also studies reported contrasting results that rTMS does not confer additional benefits in the treatment of hemiplegia [11–14], with some studies even reporting mild functional regression after rTMS treatment [15, 16]. Moreover, recent reviews on the clinical efficacy of rTMS in the treatment of stroke hemiplegia did not reach a consistent conclusion, especially in patients with severe hemiplegia or at chronic stage [13, 17–20]. According to the Clinical Application Guidelines for rTMS (2018) [21], level A evidence supports the use of low-frequency rTMS on contralesional M1, and level B evidence suggests application of high-frequency rTMS on ipsilateral M1 in patients with sub-acute stroke. However, there is no clear recommendation for rTMS stimulation parameters in patients with chronic stroke. Therefore, this calls for high quality evidence that will be used to develop an optimal rTMS protocol for chronic stroke patients with severe hemiplegia.

The interhemispheric inhibition (IHI) model, which is commonly used as the fundamental theory for standardized rTMS treatment, was first proposed by Murase in 2004 [22], which suggests that the bilateral primary motor cortexes (M1) are connected through the corpus callosum in a balance of competing inhibition in healthy individuals. However, the IHI balance is disturbed after the onset of stroke, thereby leading to over activation of the contralesional hemisphere. Specifically, damage to the affect hemisphere weakens its inhibitory effect on the contralesional hemisphere, ultimately resulting in abnormally increased excitability of cM1. Notably, the over activation of cM1 will in turn inhibit the affect M1, causing a vicious circle. The disrupted balance between bilateral M1 will eventually hinder the motor output and impede the recovery of upper limb motor function in stroke survivors. Ideally, according to the standardized treatment protocol [19, 20], high-frequency rTMS is able to increase the excitability of affected M1, whereas low-frequency rTMS is capable of reducing the excitability of cM1, thereby enabling the disrupted IHI to reach a new equilibrium. However, some studies have found that the standardized rTMS protocol is not suitable for all stroke patients [11, 12]. It is worth noting that patients with severe hemiplegia seem to be less suitable for this standardized rTMS protocol [19]. This has led to some researchers questioning the value of classic IHI imbalance theory in clinical practice. Pino et al. [23] proposed the bimodal balance-recovery model in stroke recovery, which suggests that determining the mode of recovery to be adopted depends on the degree of damage to the affected hemisphere, namely structural reserve. For example, in patients with less damage (higher structural reserve), the classic IHI imbalance mode will dominate the recovery process, and the increased activation of contralesional hemisphere will not be conducive to motor recovery. In such cases, standardized low frequency rTMS of cM1 can exert therapeutic effects by inhibiting this abnormal excitability. In contrast, for patients with low structure reverse, the recovery process will be dominated by the compensatory mode because the increased motor cortex excitability on the unaffected side will be conducive to rehabilitation. In this case, the standardized low-frequency rTMS protocol might not be effective, and thus applying high-frequency rTMS on cM1 is a better option. Taking these two recovery models together, we believe that if we can identify the cut-off of structural reserve, we might be able to evaluate the real effects (inhibitory or compensatory) of the contralesional hemisphere on motor output. This will aid in selecting the optimum rTMS stimulation strategy on contralesional hemisphere (low frequency or high frequency).

In addition, studies should clarify whether the cM1 should be used as the only target brain area on the contralesional hemisphere in the compensatory model. According to the concept of “segregation” in classic brain research, different regions of the cerebral cortex have relatively different functions and each region is responsible for a specific functional task. In fact, even for the simplest functional tasks such as reaching, a wide range of different brain regions need to interact and coordinate to form a network to complete these tasks, a process referred to as “functional integration” [24]. Therefore, to complete a motor task, secondary motor areas like premotor cortex (PMC), supplementary motor area (SMA), cingulate motor area (CMA), and other moto-related brain regions may also be involved along with the primary motor cortex. However, most rTMS studies in stroke rehabilitation have only focused on M1. When the corticospinal tract (CST) is severely damaged, it is possible that the brain reorganization pattern may not only be associated with M1; the secondary motor cortex closely linked to M1 may also be involved [25].

Against this background and based on our previous studies [24–30], we propose that PMC might play a key role beyond M1. This can be attributed to several reasons. First, homologous regions of bilateral hemispheres are connected through the corpus callosum, and the connections between bilateral PMC form the largest proportion of white matter fibers of the corpus callosum, rather than between bilateral M1 [26]. This provides the structural basis for the existence of IHI in PMC as it is in M1. Thus, the interaction between bilateral PMC in stroke recovery is worth more attention. Second, PMC is composed of the dorsal part (PMd) and the ventral part (PMv). Previous studies revealed that PMd was involved in the coordination and control of the complex movements of hands [27]. Uncrossed corticospinal ventral tracts originating ipsilateral from PMd (approximately 10–20% of CST) were also found in animal studies [28]. Moreover, PMd is associated with the reticulospinal tract and the red nucleus spinal tract emanating from the brain stem, mainly inculcating the ipsilateral proximal limb [29]. This implies that cPMd may play a compensatory role in promoting motor functional recovery through the ipsilateral inculcating pathway. Third, we have previously conducted a resting-state fMRI study in chronic stroke patients, and found that the betweenness centrality (BC) of cPMd in the stroke group was significantly lower than that in the healthy group. In addition, the BC value of cPMd was significantly positively correlated with the score of the upper limb motor function on the ipsilateral side. These results suggest that the more compensatory effects cPMd provide, the better the motor prognosis for severe stroke patients [30]. Based on the above three points, we hypothesized that cPMd may play a role as a potential target brain area for promoting brain reorganization in rTMS therapy.

Currently, only sporadic reports of high frequency rTMS on cPMd in stroke treatment exist. Johansen-Berg et al. [31] stimulated the cPMd of stroke patients with mono-pulse TMS, and found a negative correlation between the stimulus intensity and the lateral index of PMd activation in task-based fMRI, as well as a positive correlation to the hemiplegic hand motor function score. This suggests that TMS is capable of activating excitatory effects on cPMd, and facilitating the motor recovery of upper limb and hand in stroke patients. In addition, Bestmann et al. [32] found that stroke patients with more severe dysfunction demonstrated more activation in cPMd and the posterior part of M1 (BA4p) on the affected side when a single high-frequency TMS stimulated cPMd. This suggests that exciting cPMd with single high-frequency TMS may cause the activation points of the affected hand projection cortex to shift backward, thereby promoting functional recovery. A recent randomized sham-controlled crossover study published in 2017 compared the effects of single-pulse low-frequency TMS on M1, high-frequency TMS on cPMd, and sham stimulation in 15 stroke patients. The results showed that treatment using single-pulse high-frequency TMS on cPMd resulted in a greater improvement than using single-pulse low-frequency TMS on cM1 in severely affected patients [33]. However, these evidences were all based on cross-sectional studies, and longitudinal treatment studies on single-pulse high-frequency rTMS of cPMd are still lacking. Moreover, most of the existing studies utilized single-mode fMRI analysis, which could not thoroughly demonstrate the brain network reorganization patterns of rTMS in promoting functional recovery in stroke patients. Therefore, future studies should use multimodal fMRI (structural, functional, and effective network) to elucidate the mechanisms of this new protocol of rTMS in stroke rehabilitation from multiple perspectives of neural repair and remodeling.

### Aims and hypotheses

This study will conduct a longitudinal, single-blinded, randomized controlled trial to verify the clinical effects of different protocols of rTMS therapy on functional recovery of chronic subcortical stroke patients with severe hemiplegic upper limb. To the best of our knowledge, this is the first study to compare the effects of low-frequency rTMS on cM1 and high-frequency rTMS on cPMd. We expect that the findings will provide vital information about the optimal rTMS strategy. Multimodal fMRI scanning and electrophysiological measurements will be performed longitudinally to elucidate the underlying neural mechanism of brain reorganization after different parameters of rTMS therapy. We hypothesize that high-frequency rTMS on cPMd might be the optimal protocol for motor functional recovery in chronic subcortical stroke patients with severe hemiplegia. This clinical effect may be attributed to the specific remodeling patterns of structural, functional, or effective connectivity of homologous or allogeneic cortexes within or between hemispheres through multimodal neural imaging analysis. Furthermore, joint analysis of the clinical and neuroimaging results might identify the cut-off of structural reserve that determines the application of high/low frequency rTMS on contralesional hemisphere to achieve maximum functional recovery of the paretic upper limb.

## Methods/design

### Study design

**Fig. 1:**
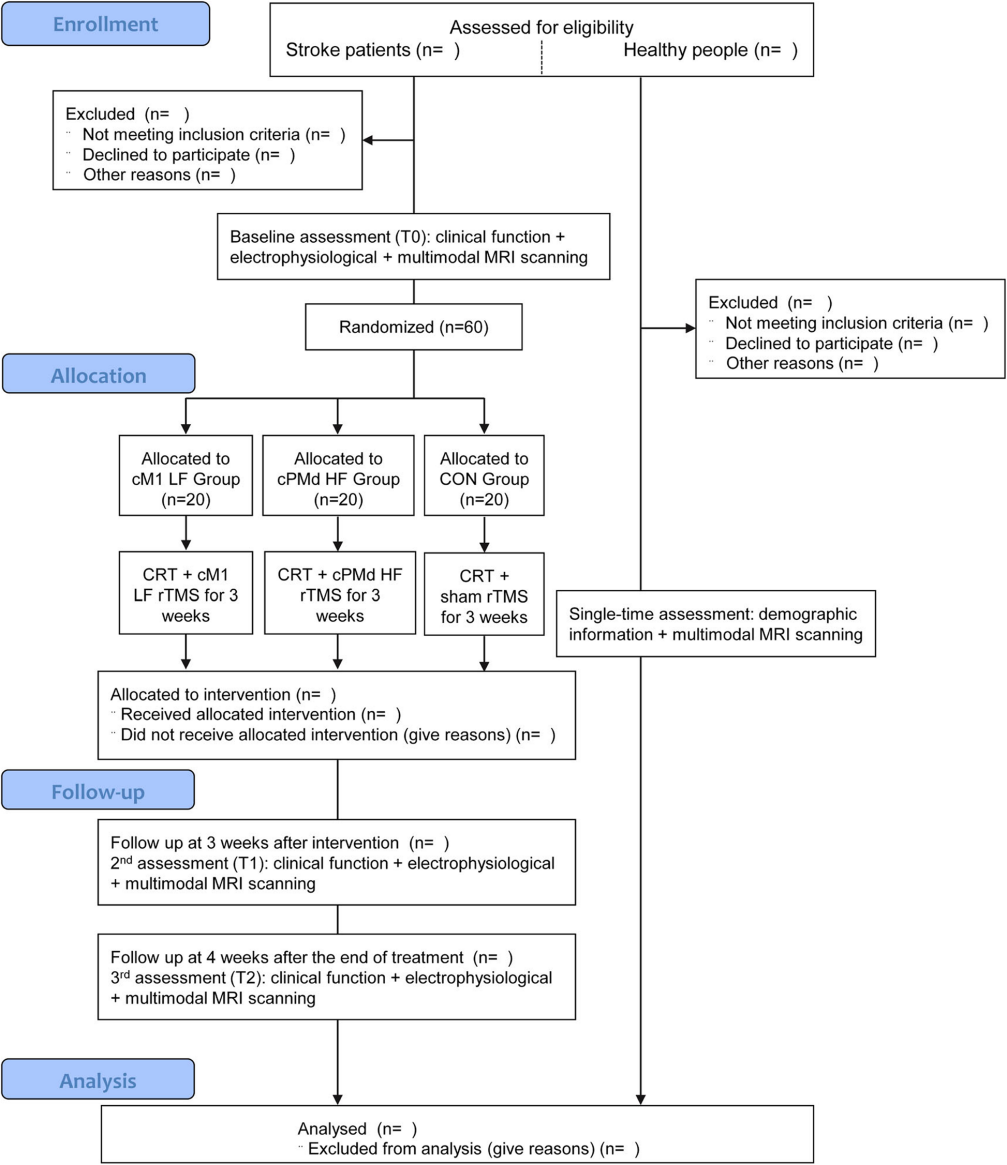
Flow chart showing the study design. CRT, conventional rehabilitation training; cM1, contralesional primary motor cortex; cPMd, contralesional dorsal premotor cortex; LF, low frequency; HF, high frequency; rTMS, repeated transcranial magnetic stimulation

The study will be conducted at Huashan Hospital, Fudan University and The Third Rehabilitation Hospital, Shanghai. Signed informed consent will be obtained from all participants prior to the study. Briefly, chronic (onset time > 3 months) subcortical stroke patients with severe hemiplegia will receive conventional rehabilitation treatment and three different protocols of rTMS intervention: low-frequency rTMS of cM1, high-frequency rTMS of cPMd, and sham stimulation. Next, multimodal fMRI scanning (DTI, rs-fMRI, and tb-fMRI), functional assessments, and electrophysiological assessments will be performed before intervention (T0), 3 weeks after intervention (T1), and 4 weeks after the end of treatment (T2), respectively. Figure 1 shows the flow-diagram of the study design.

Patients will be recruited from Huashan Hospital Fudan University and the Third Rehabilitation Hospital of Shanghai. The legal rights and interests of them will not be violated during the study. For the intervention, subjects’ privacy will be fully respected and their personal information will be kept strictly confidential.

### Inclusion and exclusion criteria

A total of 60 eligible patients will be recruited from Huashan Hospital, Fudan University. The inclusion and exclusion criteria are listed in Table 1.

**Table 1:** Inclusion and exclusion criteria

Criteria	Description
Inclusion criteria	➤ First-ever stroke ➤ Subcortical stroke diagnosed by head CT or MRI ➤ Clear consciousness and stable vital signs ➤ 18–80 years old ➤ 3–12 months since stroke onset ➤ Unilateral hemiplegia, Brunnstrom stage of affected hand ≤ IV ➤ Right-handed
Exclusion criteria	➤ Severe speech, attention, hearing, vision, sensation, intelligence, mental, or cognitive impairments (MMSE score < 27) ➤ Severe spasticity (Modified Ashworth Spasticity Scale > 2) or pain (Ten-point Visual Analog Scale > 4) of the affected side ➤ Bone, joint, and muscle diseases, other serious nervous system diseases, malignant tumors, and serious heart, lung, liver, and kidney damage ➤ Still enrolled in any rehabilitation or drug studies ➤ Metal implant, magnetic sheet, or cardiac pacemaker ➤ Alcohol or drug addiction ➤ Epilepsy

### Randomization and grouping

The 60 eligible stroke patients will be randomly assigned to three groups with 20 patients in each group: low-frequency rTMS of cM1 (cM1-LF) group, high-frequency rTMS of cPMd (cPMd-HF) group, and sham-stimulation control (CON) group. The random sequence will be created using STATA 12.0 software (StataCorp, Texas, USA) by a professional third-party statistician from Fudan University. The randomization list will only be known by this research coordinator and will be concealed from other study personnel. However, the research coordinator will not participate in recruitment, subsequent intervention and assessment, and final data analysis. In addition, 20 healthy people will be recruited into the healthy control (HC) group. The HC group will be matched with stroke groups in terms of age, gender, and education.

### Blinding

This trial will utilize a randomized, patient-and assessor-blinded, parallel-group design. The patients, assessors, and statisticians will not be aware of the group assignment. They will only be informed of the group’s identification (group A, group B, and group C) but not be aware of which group is the cM1-LF, cPMd-HF, and CON group. Physicians will be divided into two groups: one group responsible for screening and assessing patients, and the other group responsible for applying rTMS intervention. Notably, the former will be blinded to group assignment, but the later cannot be blinded since applying rTMS over different stimulation sites implies the assignment.

### Conventional rehabilitation intervention and rTMS intervention

In addition to the routine pharmacotherapy, the three groups of patients will undergo 40 min of conventional rehabilitation, including physical therapy of upper limb function (PT) and daily living ability training (OT), once each day, 5 days a week, for a total of 3 weeks. Moreover, all stroke patients will receive the same dose of other routine rehabilitation treatments such as biofeedback electrical stimulation, acupuncture, and massage for 3 weeks.

**Fig. 2:**
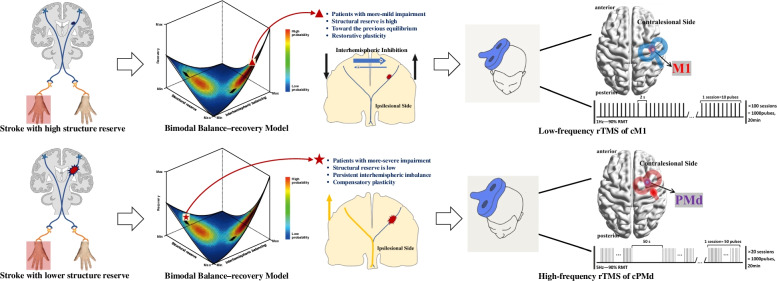
Bimodal Balance-recovery Model for rTMS protocols in this study. Bimodal Balance-recovery Model for rTMS protocols in this study. cM1, contralesional primary motor cortex; cPMd, contralesional dorsal premotor cortex; rTMS, repeated transcranial magnetic stimulation; RTM, resting motor threshold

For rTMS intervention, ① patients in the cM1-LF group will be treated as follows: 100 sessions of 1 Hz rTMS at 90% resting motor threshold (RMT) to cM1 (at the optimal stimulus point with the largest and most consistent MEP), 10 pulses per session with a 2-s interval between sessions, totaling 1000 pulses (20 min); ② patients in the cPMd-HF group will be treated as follows: 20 sessions of 5 Hz rTMS at 90% RMT to cPMd (2 cm in front and 1 cm in the inner side of the optimal stimulus point of M1), 50 pulses per session with a 50-s interval between sessions, totaling 1000 pulses (20 min) (Fig. 2); and ③ patients in the CON group will be treated with the same parameters as cM1-LF group (including location, stimulation frequency, and time), however, the coil will be placed perpendicular to the surface of the skull to emit the same stimulating sound as the cM1-LF group but without effective stimulation. All patients in the three groups will receive rTMS intervention once a day for 5 days a week, for 3 weeks.

If any patients experience headaches, nausea, or changes in their condition throughout the interventions, we will terminate the interventions and provide timely medical assistance. Adverse events will also be reported to the ETHICS committee.

### Outcome measures

All patients will receive clinical function assessment, electrophysiological measurement, and multimodal fMRI scanning at all time points. Healthy subjects will only receive fMRI scanning scan at T0. The outcome measure timing is listed in Table 2.

**Table 2:** Outcome measure timing

Groups	cM1-LF, cPMd-HF & CON group			HC group		
Time point	T0	T1	T2	T0	T1	T2
Clinical function assessment	○	○	○			
Electrophysiological assessment (MEPs, RMT)	○	○	○			
MRI scan	○	○	○	○		

#### Clinical function assessment and electrophysiological assessment

Clinical function assessment and electrophysiological measurement will be performed at T0, T1, and T2 in Huashan Hospital, Fudan University. The level of motor function will be assessed based on the Brunnstrom stage. Manual muscle test (MMT) and modified Ashworth scale (MAS) will be used to assess muscle strength and muscle tone of the limbs, respectively, whereas the national Institutes of Health Stroke Scale (NIHSS) will be used to assess the severity of neurological deficits in stroke. In addition, Fugl-Meyer Upper Limb Motor Function Rating Scale (FM-UL), Action Research Arm Test Scale (ARAT) and Wolf Motor Function Test Scale (WMFT) will be used to assess the motor function of the affected arm and hand. Activities of daily Living (ADL) will be evaluated using the Modified Barthel Index (MBI).

For electrophysiological evaluation, single-pulse TMS (YRD CCY-4, Yi Ruide Company, Wuhan, China) will be used to assess bilateral hemispherical MEP using a 8-figured coil with an external wing of 7 cm. We will first determine the optimal stimulus point, the location on the scalp with the largest and most consistent MEP in the contralateral Abductor Pollicis Brevis (APB), from which the subsequent neurophysiological assessments will be performed. The resting motor threshold (RMT), which is the minimal stimulus intensity required to evoke MEPs with amplitudes of ≥50 μV in at least five out of 10 consecutive trails, will be determined as the amplitude reference. MEP amplitude and latency, i.e., the peak to peak amplitude and the time period between stimulation onset and start of the largest MEP, will be measured and recorded. In instances where MEP will be recorded in the affected-side APB, it will be denoted as MEP (+), and if multiple measurements fail to yield a MEP, it will be denoted as MEP (−).

#### fMRI scan

The multimodal fMRI scanning will be performed for patients at T0, T1, and T2, and at T0 for healthy subjects using a Siemens Trio 3 Tesla MRI scanner (Siemens, Erlangen, Germany) in Shanghai Key Magnetic Resonance Laboratory of East China Normal University.

- ① Motor tasks

**Fig. 3:**
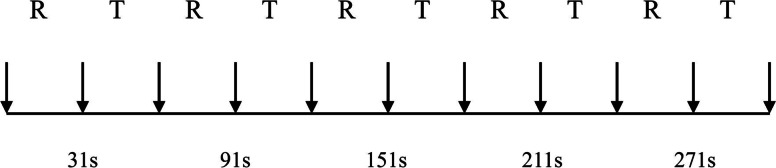
Block design for tb-fMRI. Block design for tb-fMRI. A total of 10 blocks include 5 REST and 5 TASK are adopted for the tb-based fMRI. Each block last for 30 s. R, REST; T, TASK

A block design will be adopted for the task-based fMRI scanning. In each run, there will be five blocks in baseline and five blocks in task state, and each block will last 30 s (as shown in Fig. 3). In addition, each run will be preceded by a 6 s empty scan, thus, the total time for each run will be 5 min and 6 s.

In each task state block, the experimenter will make a passive clench motion of fingers on the subject after receiving the “close” command. Next, the experimenter will make a passive stretching motion of fingers on the subject upon hearing “open”, with a frequency of about 1 Hz and the maximum range. Each subject will be asked to complete two groups of exercise tasks: left-hand passive clenching and right-hand passive clenching. Notably, each subject will receive relevant stimulus task training and guidance prior to fMRI.

- ② Data acquisition

The multimodal scans will be performed in accordance with the parameters listed in Table 3. TE is the time of echo, TR is the time of repetition, MPRAGE is magnetization prepared rapid acquisition with gradient echo sequence, TSE is turbo spin echo sequence, and SS-EPI is single shot gradient echo imaging sequence. In addition, for DTI, the diffusion weighted images will be acquired along 10 diffusion directions with b = 0 and 64 different diffusion directions with b = 1000 s/mm2.

**Table 3:** Parameters of multimodal MRI

Modal	TR (ms)	TE (ms)	Flip angle (°)	Number of slices	Slice thickness (mm)	Slice spacing (mm)	Sequence
3D high resolution T1-weighted image	2530	2.98	7	192	1	0.5	MPRAGE
T2-weighted image	6000	95	120	30	5	0	TSE
rs-fMRI	2000	30	90	30	4	0.8	SS-EPI
tb-fMRI	3000	30	90	30	5	0	SS-EPI
DTI	8500	63	90	75	2	0	SS-EPI

### Data processing and analysis

#### MRI preprocessing

MRI data will be preprocessed using SPM 8 software. The data of patients with left hemisphere lesions will be flipped along the midsagittal plane to ensure that all the patients have “virtual” right hemisphere lesions. SPM 8 will also be used for BLOD fMRI data preprocessing. Slicing timing, realignment, normalization, and smoothing will be performed on the BLOD fMRI data. Next, the data with translation parameter greater than ±2.5 mm or rotation parameter greater than ±2.5 degrees will be removed. Finally, the diffusion toolkit software (http://www.trackvis.org/dtk/) will be used to pre-process the DTI data.

#### Rs-fMRI

A total of 90 network nodes will be defined according to Anatomical Automatic Labeling (AAL) atlas. Pearson correlation analyses will then be performed on the preprocessed RS fMRI data, which will result in one 90 × 90 correlation matrix representing the functional connection between each two network nodes. A threshold will be used to binarize the matrix, and all correlation coefficients greater than the threshold will be assigned 1, otherwise 0. Topology attributes, including global network efficiency (Eg), local network efficiency (El), network betweenness, and clustering coefficient will be calculated for each patient based on the correlation matrix.

#### Tb-fMRI

For each patient, the preprocessed task fMRI data will be used to construct a general linear model (GLM) according to the experimental design, with a regression on the head-motion parameters.

#### DTI

The bilateral posterior limb of internal capsule (PLIC), bilateral cerebral peduncle, and corpus callosum of each patient will be delineated manually by two radiologists. For each patient, the common part of the results recorded by the two radiologists will be adopted as the regions of interest (ROIs). The mean fractional anisotropy (FA) will be calculated with all the ROIs for each hemisphere. Notably, FA asymmetery index (FA AI) is defined by the following formula:

$$FA\ AI=\frac{\left({FA}_c-{FA}_a\right)}{\left({FA}_c+{FA}_a\right)}$$

where FAc is the FA of the contralesional hemisphere and FAa is the FA of the affected hemisphere. FA AI can be used to quantitatively evaluate the structural integrity of CST and corpus callosum.

### Statistical analysis

#### Sample size calculation

The sample size for this study was calculated using SPSS 21 software based on a previous study [34]. The mean score of FM-UL assessment in patients who received low-frequency rTMS treatment was 30 at baseline and increased to 43 after intervention, with a common standard deviation of 17. Furthermore, we assumed that the study would have a dropout of 30%, and thus the final target sample size was 60 participants, with 20 assigned to each group when setting a power of 80% and a significance level of 0.05.

#### Group-level analysis

All statistical analyses will be performed using SPSS software (SPSS 25.0, SPSS Inc., Chicago, Illinois). The intention-to-treat principle will be used for the analysis of functional and electrophysiological data. One-way analysis of variance (ANOVA) will be used to compare the demographic characteristics and baseline assessment (T0) of all outcomes among groups. To compare the treatment effects of different rTMS protocol, a two-way mixed design ANOVA with between-subject factor “Group” and within-subject factor “Time” will be performed for the clinical and electrophysiological changes. Moreover, Bonferroni correction will be employed for post hoc multiple comparisons. *P* < 0.05 will be considered statistically significant for all analyses.

For rs-fMRI and DTI data, a two-way mixed design ANOVA will be performed, with between-subject factor “Group” and within-subject factor “Time” on the data generated in the data processing step, including Eg, El, network betweenness, clustering coefficient, and FA AI. For tb-fMRI data, a whole brain paired T-test will be performed between the task state blocks and baseline blocks in the FC group, and the voxels whose t value is higher than the threshold will be regarded as the activated brain region. For cM1-LF, cPMd-HF, and CON groups, a two-way mixed design ANOVA, with between-subject factor “Group” and within-subject factor “Time” will be performed within the former activated brain region. The most significant point of “Group” × “Time” effect in the activated brain region will be used as the center to construct spherical balls with a radius of 6 mm, and the spherical balls will be regarded as the ROIs. Finally, to obtain the effective connectivity of each patient at different time points, the average time series of all voxels in each ROI will be subjected to dynamic causal model (DCM) analysis.

#### Correlation analysis

Pearson correlation analysis will be performed between electrophysiological parameters, MRI parameters, and clinical function scores in cM1 LF group and cPMd HF group. The statistical threshold will be set to *p* < 0.05.

### Quality control

Huashan Hospital will monitor the following aspects of the trial regularly and strictly: the work division and training for the research staff, file management, informed consent, protocol compliance, subject recruitment, filling in the CRF, intervention, quality assured system, statistical analysis, and data management.

## Discussion

Several studies conducted in recent years have confirmed that rTMS can effectively improve the motor function of stroke patients with hemiplegia [7–10]. However, negative results have also been reported, with some studies even observing functional regression in some patients [11–16]. These evidences suggest that the standardized rTMS protocol based on the IHI imbalance theory between bilateral M1 has its limitation in clinical practice [19, 20]. In chronic stroke patients with severe motor impairments, extensive damage to affected pathway of motor output hinders the effects of affected M1 in motor recovery, thereby rendering classic rTMS protocol ineffective [18]. Thus, this study will focus on cPMd based on a more recent “bimodal balance-recovery model” [23]. Evidence suggests that inhibition of cPMd directly impairs movement of the paretic extremity in severe patients [33], and the betweenness centrality of cPMd is significantly positively correlated with the motor score [35]. Therefore, we hypothesized that cPMd is more likely to be the key factor for functional recovery of severely affected patients. This hypothesis will be verified by conducting a randomized controlled trial, which will directly compare the effects of low-frequency rTMS on cM1, high-frequency rTMS on cPMd, and sham-stimulation. It is expected that the findings of the study will facilitate development of a new protocol of rTMS therapy for treating severely affected stroke patients.

In addition to potential clinical influence, the study will elucidate the underlying mechanism of brain plasticity though longitudinal analysis of multimodal fMRI data scanned before and after the intervention, and during the follow up. It is worth noting that rest-state fMRI will provide information about the changes of brain networks, task-based fMRI will display the brain activation model of specific task instantly, and DTI will provide insights into the remodeling of motor pathway. Collectively, the results will help us to further understand the neural effects of different protocols of rTMS therapy in stroke rehabilitation. Furthermore, the joint analysis of neuroimaging data and functional changes will indicate the cut-off that separates mildly from severely affected patients, ultimately guiding application of tailored stimulation parameters for rTMS (i.e. low-frequency rTMS on cM1 or high-frequency rTMS on cPMd). In conclusion, this study will provide a theoretical basis for clarifying the bimodal balance-recovery model of stroke, provide biological markers for predicting the functional prognosis in chronic stroke patients after rTMS therapy, and provide a strategy for individualized rTMS treatment for stroke in future studies and clinical practice.

## Trial status

This trial was registered on 12 November 2019. Patient recruitment began on 1 January 2020 and will continue until 31 December 2023. Recruitment was not completed at the time of submission.

## Data Availability

The metadata and protocol will be submitted to the clinical trial center of Huashan Hospital before June 30,2024. And the calculated results will be able to be downloaded at http://www.chictr.org.cn/showproj.aspx?proj=43686.

## Abbreviations

TMS: Transcranial magnetic stimulation
rTMS: Repeated TMS
M1: Primary motor cortex
cM1: Contralesional M1
PMC: Premotor cortex
cPMd: Contralesional dorsal PMC
fMRI: Functional MRI
NIBS: Non-invasive brain stimulation
IHI: Interhemispheric inhibition
SMA: Supplementary motor area
CMA: Cingulate motor area
CST: Corticospinal tract
BC: Betweenness centrality
CRT: Conventional rehabilitation training
MMT: Manual muscle test
NIHSS: National Institutes of Health Stroke Scale
MAS: Modified Ashworth scale
FM-UL: Fugl-Meyer Upper Limb Motor Function Rating Scale
ARAT: Action Research Arm Test Scale
WMFT: Wolf Motor Function Test Scale
ADL: Activities of daily Living
MBI: Modified Barthel Index
MEP: Motor evoked potential
APB: Abductor Pollicis Brevis
RMT: Resting motor threshold
AAL: Anatomical Automatic Labeling
GLM: General linear model
PLIC: Posterior limb of internal capsule
ROI: Regions of interest
FA: Fractional anisotropy
DCM: Dynamic causal model
